# From Inert N_2_ to Reactive Nitrate: A Comparative Analysis on Electrocatalytic Urea Synthesis via CO_2_ Coelectrolysis

**DOI:** 10.1021/acs.langmuir.6c02719

**Published:** 2026-09-17

**Authors:** Leandro A. Faustino, Susana I. Córdoba de Torresi

**Affiliations:** Instituto de Química, Universidade de São Paulo, Av. Prof. Lineu Prestes 748, 05360-050 São Paulo, SP, Brazil

## Abstract

Electrochemical urea synthesis through C–N bond formation under mild conditions has emerged as a promising alternative to the conventional energy-intensive Bosch–Meiser process, offering a sustainable pathway coupling carbon and nitrogen cycles. In this context, the coelectrolysis of CO_2_ with different nitrogen sources, N_2_ and NO_3_^–^, has attracted increasing attention. However, these routes present fundamentally distinct challenges and opportunities regarding substrate activation, intermediate formation, and product selectivity. This mini-review provides a comprehensive comparison between N_2_ + CO_2_ and NO_3_^–^ + CO_2_ coelectrolysis, focusing on the key factors that drive C–N coupling. Intrinsically limitations associated with the high-energy activation for breaking the N≡N triple bond of N_2_ and its associated low solubility, in contrast with the higher reactivity and availability of NO_3_^–^ species, were discussed. Mechanistic insights into C–N coupling pathways were critically analyzed, highlighting the role of adsorbed intermediates and competing reactions such as hydrogen evolution and side products from nitrogen-species reduction. Furthermore, recent advances regarding the catalyst design and operational conditions were evaluated in terms of their ability to enhance selectivity toward urea synthesis. Finally, we outline the current challenges and future perspectives for the rational development of next-generation catalytic systems for sustainable urea synthesis.

## Graphical abstract

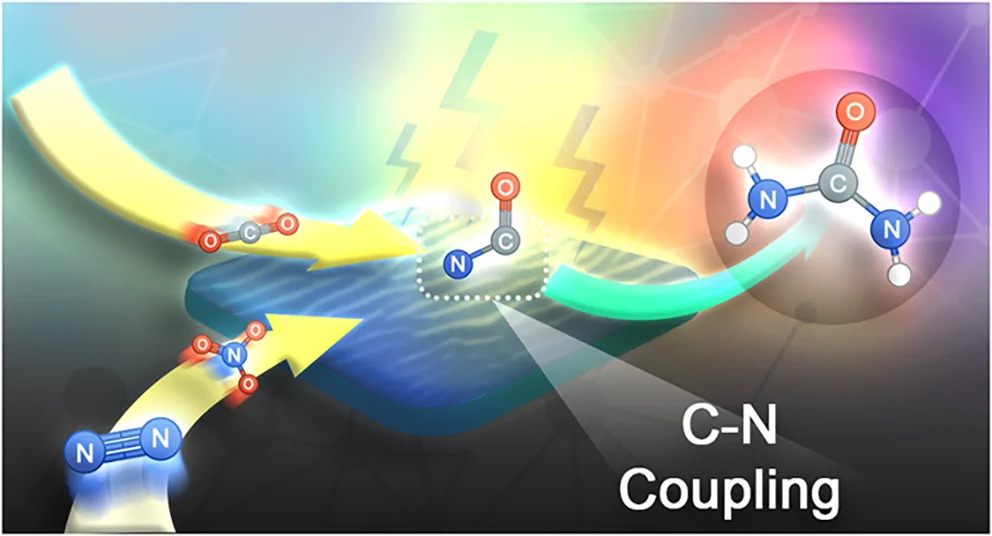

## 1 Introduction

Modern civilization relies on a wide range of energy-intensive processes that impose concurrent economic and environmental burdens. The fast growth of artificial intelligence (AI) exemplifies this trend, with its escalating computational demands driving a substantial increase in energy consumption and associated CO_2_ emissions. Recent studies indicate that training large-scale AI models requires energy inputs exceeding 1500 kWh and emissions approaching 500 tons of CO_2_.^1,2^ The growing energy demand and CO_2_ emissions are not restricted to computational ramifications. Other traditional and indispensable activities for humanity, such as the synthesis of chemical feedstocks, have also raised concerns. From data-driven technologies, such as artificial intelligence, to large-scale chemical manufacturing, modern energy-intensive activities are increasingly perturbing the global carbon cycle. Both AI development and chemical feedstock production domains reflect a unify and systematic constraint: the fast increase in energy demand and its inherent carbon emissions result in the need for renewable and sustainable processes.^3,4^

Within this context, the development of sustainable routes for chemical synthesis has become imperative; an example of this is the urea synthesis process. Urea is an important chemical feedstock applied in fields such as polymers, adhesives, ruminant additives, and pharmaceutical components, and it is a cornerstone chemical for the fertilizer industry.^5–9^ Traditionally, the urea synthesis process (Bosch–Meiser process) is closely coupled with the ammonia synthesis process (Haber–Bosch process).^10^ In the Haber–Bosch process, N_2_ reacts with H_2_ to produce NH_3_ as a product. Such H_2_ is necessary to promote N_2_ hydrogenation and commonly comes from nonrenewable sources like methane reform, which is an aggravating factor for the process.^11^ Afterward, in the Bosch–Meiser process, NH_3_ previously produced in the Haber–Bosch process reacts with CO_2_ to produce ammonium carbamate, which is further converted into urea.^12^ Despite both processes having been extensively employed, several drawbacks have raised concerns; for example, as in these catalytic processes, high temperatures and pressure are employed.^9^ In addition to the harsh conditions that make these processes costly, such processes are also responsible for the large amounts of CO_2_ emissions responsible for the greenhouse effect.^11,13^ Moreover, given that urea is a key chemical feedstock for the fertilizer industry and, consequently, for the global food supply chain, recent global events, such as the COVID-19 pandemic and geopolitical tensions (*e*.*g*., between the United States and Iran), have further exacerbated the limitations associated with its current production routes.^14^

Based on the aforementioned aspects, a clear dilemma emerges: on the one hand, the high urea demand is unavoidable; on the other, its current production method is closely related to the environmental crisis and geopolitical tensions.^15^ In this context, the development of alternative processes to replace at least partially the Bosch–Meiser process is urgently needed to mitigate such drawbacks.^16,17^

Urea synthesis through electrocatalysis has attracted attention due to its different advantages compared with the traditional Bosch–Meiser process. Such attention is partially related to the mild conditions of temperature and pressure employed, and the possibility of using H_2_ from renewable sources through the hydrogen evolution reaction (HER) from water electrolysis.^18–20^ Moreover, the possibility of using CO_2_ as a substrate, reaching zero-carbon emission, makes the process even more attractive.^21–24^

The first report in the literature, to the best of our knowledge, regarding urea electrosynthesis was performed by Furuya and colleagues in 1995, demonstrating that the coreduction of CO_2_ in the presence of NO_3_^–^/NO_2_^–^ using Cu-loaded gas diffusion electrode leads to the production of carbon-rich and nitrogen-rich species, including urea.^25^ Two decades later, in 2016, Kayan and Köleli went even further and demonstrated that CO_2_ reduction in the presence of N_2_ using conducting polymer electrodes also led to urea formation.^26^ Beyond introducing alternative routes for urea synthesis distinct from conventional thermocatalytic approaches, these studies further demonstrate the feasibility of producing urea via C–N coupling, in a single electrocatalytic step, in contrast with the Bosch–Meiser process (Figure 1).

**Figure 1:**
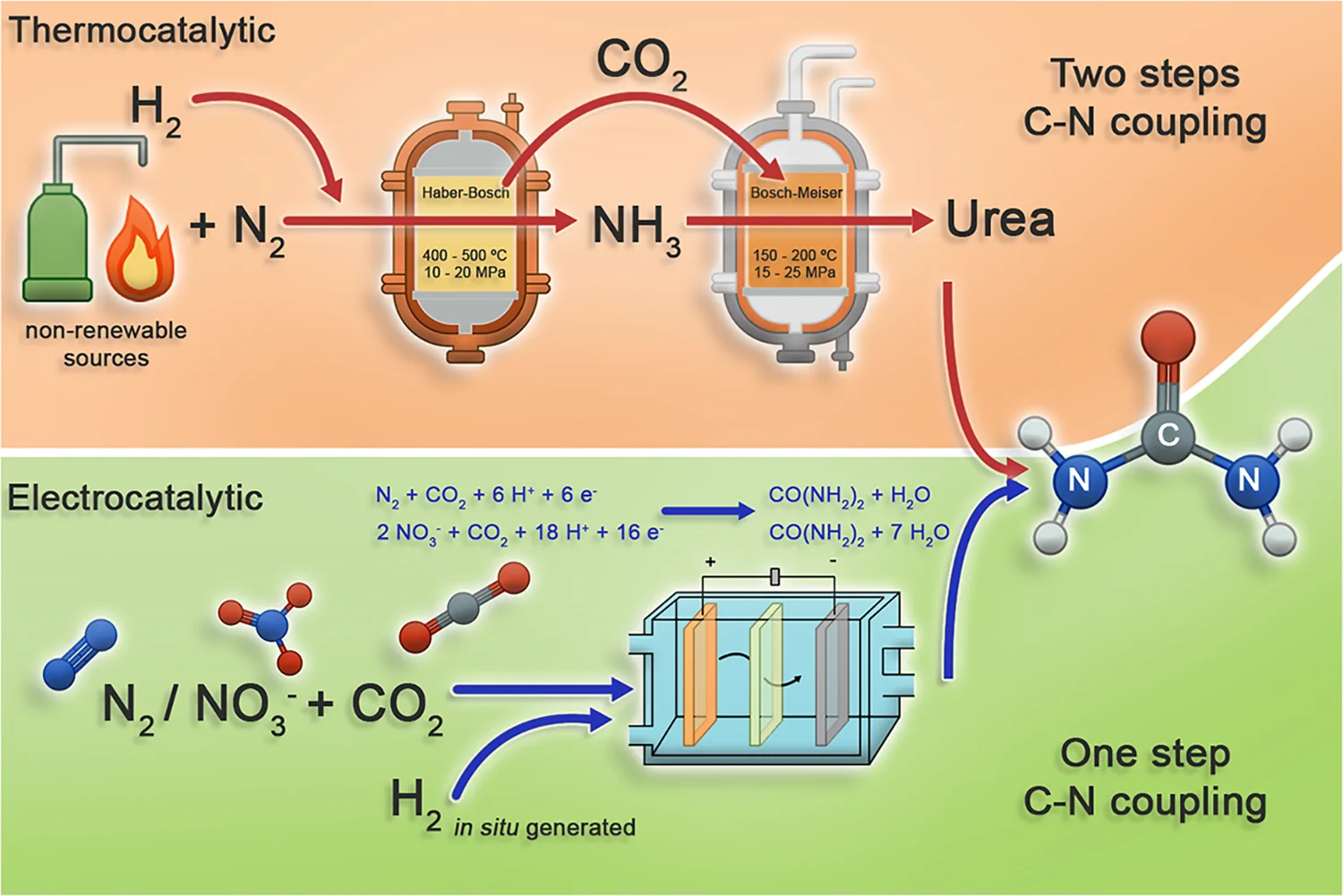
Schematic representation of urea synthesis through conventional thermocatalytic and electrocatalytic processes starting from N_2_, NO_3_^–^, and CO_2_.

In view of this, significant efforts have been devoted in recent years to urea electrosynthesis, focusing on different approaches which include investigation of different catalyst architectures, electrolyte compositions, and nitrogen sources, such as N_2_ and NO_3_^–^, in the presence of CO_2_.^27–29^ In this context, this Mini-Review provides a critical comparison of N_2_ + CO_2_ and NO_3_^–^ + CO_2_ coelectrolysis routes, highlighting their respective advantages, drawbacks, key challenges, and inherent limitations.

It is worth noting that several recent reviews have already provided valuable overviews of this field, and it is important to clarify how the present work differs from those. Li and colleagues systematically cataloged electrocatalyst families, *e*.*g*., carbon-based, metallic, single-atom, and diatomic materials across the full set of nitrogen sources like N_2_, NO, NO_2_^–^, and NO_3_^–^ for the individual CO_2_RR, N_2_RR, NO_2_^–^RR, and NO_3_^–^RR (RR, reduction reactions) half-reactions.^30^ In the work of Kohlhaas and colleagues, the authors focused on reactors and process-engineering bottlenecks, such as H-cell versus flow cells, gas diffusion electrode (GDE) configurations, membrane sections, and techno-economic scale-up, that are largely common across nitrogen sources.^31^ Furthermore, in the revision of Muhyuddin and colleagues, they examined in depth the reaction mechanisms and *in situ*/*ex situ* detection methodologies specific to the NO_3_^–^ + CO_2_ route.^32^ These valuable contributions have largely been structured around catalyst chemistry, reactor engineering, or individual nitrogen feedstock. Yet, none have placed N_2_ and NO_3_^–^ in direct comparison as the central framework for discussion. A critical gap therefore remains in the literature: a mechanistic, side-by-side assessment of these two extensively investigated nitrogen feedstocks, whose distinct chemical and thermodynamic characteristics give rise to fundamentally different reaction barriers. Rather than organizing the discussion solely according to the catalyst class, this comparative perspective focuses on the physicochemical origins of these barriers, encompassing adsorption energetics, bond dissociation energies, and the complexity of proton-coupled electron transfer pathways. The present comparative mini-review addresses this gap by establishing an explicit, quantitatively supported framework (Section 4 and Table 3) for evaluating the intrinsic advantages and limitations of N_2_ and NO_3_^–^, thereby providing a rational basis for selecting the most suitable nitrogen feedstock according to the target application and desired performance metrics.

## 2 Electrocatalytic C–N Coupling from N_2_ and CO_2_

As highlighted above, the continuously rising CO_2_ emissions, while central to the environmental crisis, paradoxically render this molecule an abundant and readily accessible carbon feedstock. Similarly, N_2_, although it is not considered a pollutant, has an overwhelming presence in the atmosphere (∼78%), making it an inexhaustible nitrogen source.^33,34^ From this perspective, the direct utilization of N_2_ and CO_2_ emerges as an ideal scenario for promoting C–N coupling toward sustainable urea electrosynthesis; however, several challenges are associated with it. The main challenges considering the N_2_ and CO_2_ as substrates remain in three major pillars: (i) adsorption, (ii) high-energy dissociation, and (iii) parallel side reactions.

The first challenge to be considered in electrochemical C–N coupling for urea electrosynthesis lies in the inherent weak and competitive adsorption of N_2_ and CO_2_. The N_2_ and CO_2_ molecules are geometrically linear, not easily polarized, and do not produce moment dipoles to be adsorbed over the catalyst surface, resulting in weak adsorption.^30,35,36^ Moreover, considering the distinct characteristics of both substrates, it leads to a competition for the active catalytic sites of both species. These factors combined make it difficult to achieve a controlled surface in which both species are spatially adsorbed and activated to lead to C–N bond formation and consequently urea. To overcome these challenges, the design of catalysts that promote colocalization and selective activation of both species has gained increasing attention, resulting in complex architectures. Some of the examples that can be cited consist of the use of single-atom catalysts (SACs), which have a modulated charge distribution ideal for the adsorption of both species and activation of them to lead to C–N coupling. However, such an approach still suffers from stability in operational conditions, which is a challenge.^37^ Heterojunction-based systems have been reported as a promising approach, as they can provide differentiated yet synergistic active sites facilitating the simultaneous adsorption and activation of N_2_ and CO_2_ for C–N coupling. Yuan and colleagues demonstrated these achievements using catalysts based on semiconductors, BiVO_4_, BiFeO_3_, and the heterojunction BiVO_4_/BiFeO_3_ through theoretical calculations, temperature-programmed desorption (TPD), and N_2_ and CO_2_ coelectrolysis.^38^ In theoretical calculations, the authors demonstrated that each individual semiconductor presents different and positive free energy adsorption for each gaseous species (Figure 2a). However, when combined to give BiVO_4_/BiFeO_3_, the free energy adsorption path becomes energetically favorable for both N_2_ and CO_2_. In the TPD experiments, the authors demonstrated such adsorption improvement for both gaseous species on the heterojunction BiVO_4_/BiFeO_3_, with larger desorption peak values compared to pristine BiVO_4_ and BiFeO_3_ (Figure 2b,c). Moreover, it was proved in the coelectrolysis of N_2_ and CO_2_, in which the individual semiconductors lead to Faradaic efficiency for urea electrosynthesis with values lower than 8%, contrasting with the obtained for BiVO_4_/BiFeO_3_ of 18%, that is, almost 2 times higher (Figure 2d).

**Figure 2:**
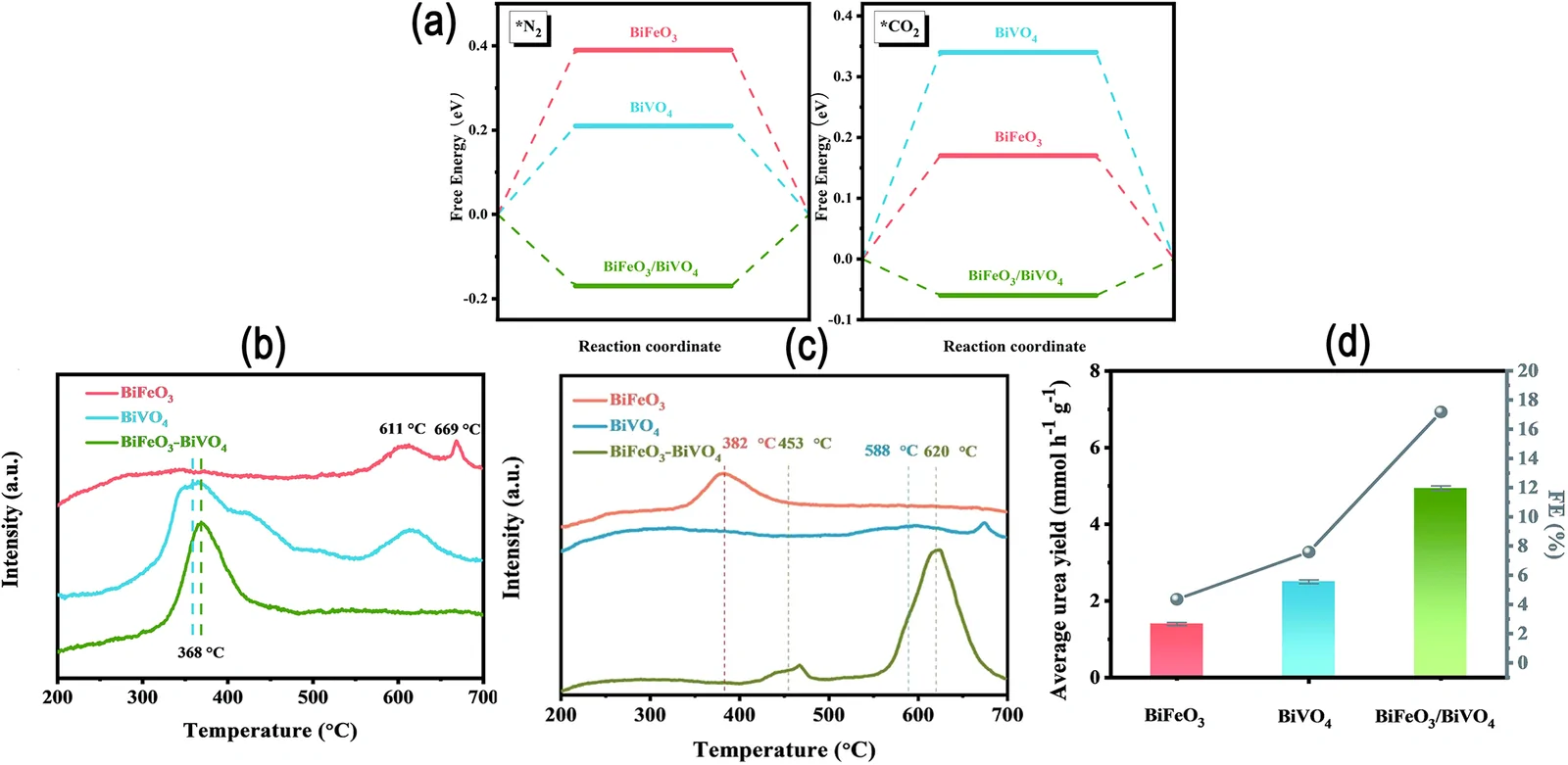
(a) Free energy diagrams for N_2_ and CO_2_ adsorption, temperature-programmed desorption of (b) carbon (CO_2_-TPD), (c) nitrogen (N_2_-TPD) spectra, and (d) average urea yield rates and Faradaic efficiencies of BiFeO_3_, BiVO_4_, and BiVO_4_/BiFeO_3_. Adapted from ref 38. Available under CC BY 3.0. Copyright 2021 Yuan, Published by Royal Society of Chemistry.

Despite adsorption being a challenge that needs to be overcome during C–N coupling, the inertness of N_2_ and CO_2_ also plays an important role. The energy activation for these gaseous species is 941 kJ mol^–1^ and 806 kJ mol^–1^ to dissociate N≡N and C=O from N_2_ and CO_2_, respectively.^39^ Moreover, in the case of N_2_ molecules, the energy gap between their HOMO–LUMO orbitals is 10.82 eV.^40^ These energies reinforce that the catalyst structure must be complex enough to overcome this inertness and then promote the activation of these species, leading to C–N coupling. Different approaches have been discussed in the literature, with catalysts based on frustrated Lewis pairs (FLPs) being important examples. Concerning the FLPs catalysts, in another study carried out by Yuan and colleagues, the authors designed structures based on [Ni_3_(BO_3_)_2_] where superficial hydroxyl groups and neighboring sites of Ni act as Lewis basic and acidic sites, respectively.^41^ The authors propose that both Lewis sites interact with the N_2_ and CO_2_ molecules through orbital interactions (Figure 3a).

**Figure 3:**
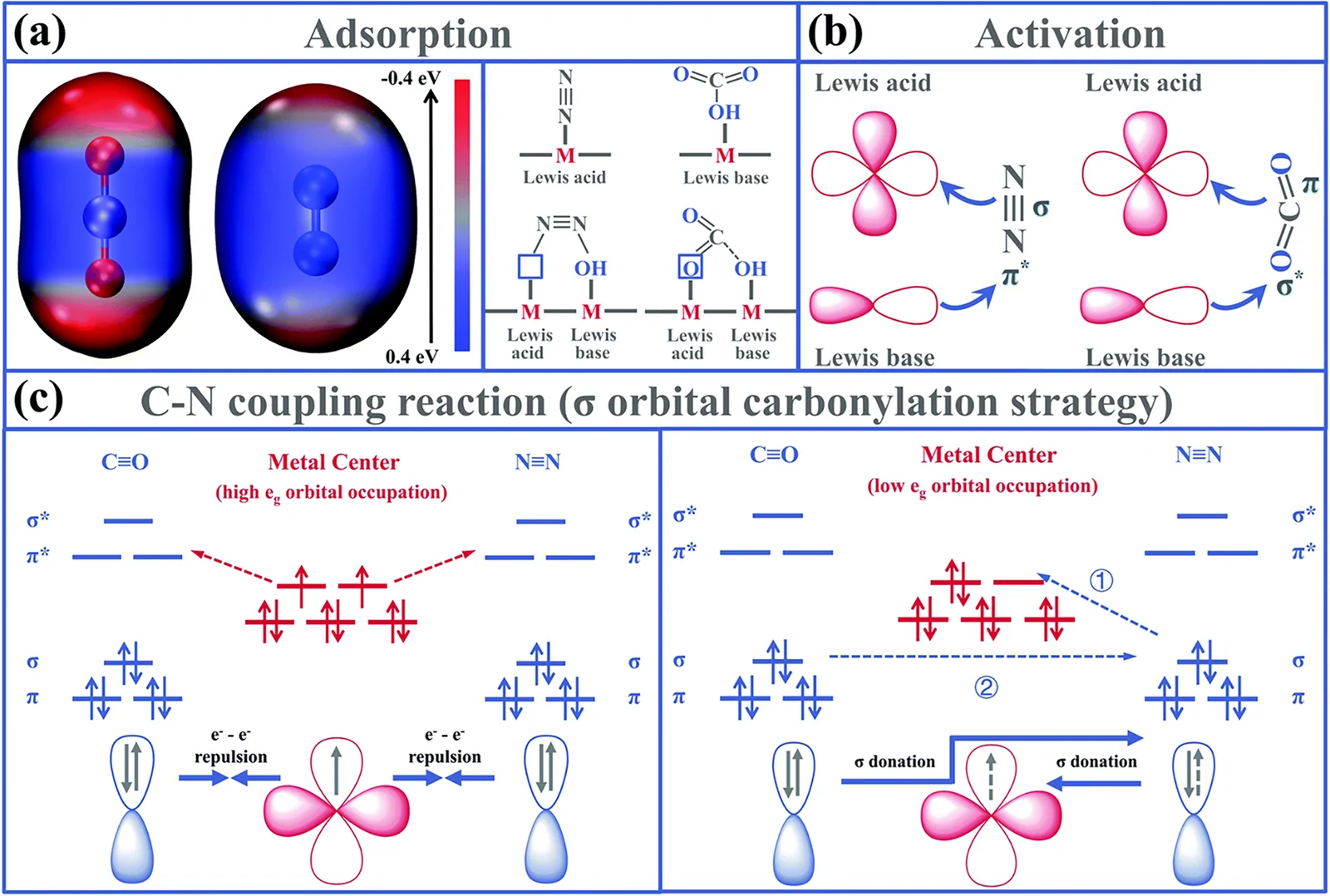
Schematic illustration of the contribution of artificial frustrated Lewis pairs in the (a) adsorption, (b) activation, and (c) C–N coupling reaction steps during the urea electrosynthesis process. Reproduced from ref 41, with permission from the Royal Society of Chemistry, Copyright 2021.

It has been pointed out that the bonding and antibonding orbitals of gaseous species interact with the empty and nonbonding orbitals of Lewis acid and base sites, respectively, resulting in the activation of the species, resulting in C–N coupling (Figure 3b). The population of antibonding orbitals of gaseous species results in the reduction of the bond order until the bonds are broken, generating intermediates like *CO and *N_2_. Since these intermediates are formed, the forward steps will then lead to C–N coupling (Figure 3c).^41^ However, the balance between acidity and basicity in the design of catalysts based on FLPs must be considered. The N_2_ activation by FLPs requires a Lewis acid center strong enough to accept the electron density from the N_2_ π* orbitals, while the Lewis base donates to the σ* orbital, weakening the N≡N bond. CO_2_ activation follows the same premise but with a geometrically distinct pathway. The challenge of simultaneous activation lies in finding a Lewis acid/base pair with moderate strength where a too weak acid site fails in the N_2_ activation, while a too strong acid may irreversibly bind CO_2_, forming stable products, leading to the poisoning of the active site.^41,42^

As previously mentioned, considering the factors like the simultaneous adsorption and activation of N_2_ and CO_2_ for the electrochemical C–N coupling, the system is prone to parallel reactions.^43^ To the best of our knowledge, until now, studies of N_2_ and CO_2_ coelectrolysis are based on the use of aqueous electrolytes facing significant challenges regarding parallel side reactions, overlapping potential windows, and the complexity of controlling multiple reaction intermediates on different steps at the catalyst surface. Concerning the high energy necessary to break down the N≡N bond, high negative potentials are typically required in a range where other reactions become dominant. In aqueous electrolyte systems, the most critical competing reaction is the hydrogen evolution reaction (HER), which is thermodynamically favored and kinetically fast.^18,44,45^ As a result, at the potential required for N_2_ activation, the production of H_2_ from water electrolysis often competes with nitrogen reduction, lowering the FE toward urea formation. Furthermore, the presence of CO_2_ introduces another layer of complexity. Generally, CO_2_ reduction proceeds at a different potential compared to N_2_ reduction, leading to a mismatch in optimal operating conditions.^46^ This creates a narrow and delicate potential window in which N_2_, CO_2_, and H_2_O can be activated concurrently to enable C–N coupling.

Another major challenge, as a result of different potentials in which the species are activated, is the control of a large number of intermediates through multiple proton-coupled electron transfer steps, which generate species such as *NNH, *N_2_H_2_, *NH_2_, *CO, and *COOH.^31^ For the C–N coupling leading to urea formation, the specific intermediate from each pathway must coexist on the catalyst surface at the right time and in the correct space and configuration to enable C–N bond formation. Small deviations in the potential can shift the reaction pathway to other products like NH_3_, N_2_H_2_, CO, HCOOH, and H_2_ instead of urea.^47^

In view of this, understanding the mechanism and intermediate species involved during N_2_ and CO_2_ coelectrolysis is an important tool to support the rational control of intermediates as well as side reactions for a more efficient C–N coupling process. On the basis of this, it is pointed out that the premise for urea electrosynthesis is preceded by ammonia formation to further C–N coupling. In fact, it reflects that two steps would be involved, that is, the N_2_ being converted into NH_3_ to further react with CO_2_ to yield urea, as in the Haber–Bosch and Bosch–Meiser processes. However, in the studies carried out by Wang and colleagues, the authors demonstrated through different techniques and labeled experiments that this premise is not true for all the cases. First, the authors investigated urea electrosynthesis using ^14^N_2_ and ^15^N_2_, and a mixture of ^14^N_2_^15^N_2_ was monitored by LC-MS/MS (liquid chromatography–mass spectroscopy/mass spectroscopy). If ammonia was one intermediate involved during C–N coupling, the product distribution expected would be CO­(^14^NH_2_)_2_ (*m*/*z* + H^+^ = 61), CO­(^14^NH_2_^15^NH_2_) (*m*/*z* + H^+^ = 62), CO­(^15^NH_2_)_2_ (*m*/*z* + H^+^ = 63), and CO­(^14^NH_2_^15^NH_2_) in the ratio of 1:2:1, respectively. Although the authors observed the product distribution of 1:0:1 and confirmed it by theoretical calculation of energies, direct C–N coupling without the formation of NH_3_ in one pot is more feasible.^39,48^ Another fact that ensures this is that in the absence of CO_2_, that is, only N_2_ present in the medium, the catalyst presents a very poor catalytic activity for ammonia yield. Such correlations indicate that only one molecule of N_2_ participates in C–N coupling, in which the N–N bond is not completely broken into intermediate species, leading to selectivity for urea synthesis. Such an example reveals the intrinsic property of the catalyst in the controlled activation of N_2_ through the modulation of intermediates in the presence of CO_2_. Regarding C–N coupling, it is clear and necessary to have a deep investigation not only restricted by the intermediates formed but also in the behavior of the catalyst structure during C–N coupling by *in situ* and *operando* techniques to stabilize such transient states. In this context, state-of-the-art *in situ* and *operando* techniques have become indispensable to bridging the gap between catalytic performance and mechanistic understanding. Among them, X-ray Absorption Spectroscopy (XAS), including XANES and EXAFS (X-ray Absorption Near Edge and Extended X-ray Absorption Fine Structure, respectively), has been widely employed to monitor the dynamics and evolution of the electronic and local atomic structure under working conditions.^49^ These techniques are particularly powerful for identifying changes in the oxidation state, coordination environment, and possible structural reconstruction processes that may occur during an electrochemical process. However, beyond XAS, complementary *operando* spectroscopies such as Raman and differential electrochemical mass spectrometry (DEMS) have also emerged as key tools to directly probe reaction intermediates, adsorbed species, and reaction products in real time, providing molecular-level insights that are otherwise inaccessible through *ex situ* characterization.^50^ Despite their proven impact on related electrocatalytic processes, to the best of our knowledge, most of the studies have concentrated on individual reactions like CO_2_ reduction, NO_3_^–^ reduction, and water electrolysis, with limited reports on C–N coupling.^51–53^

Other approaches concerning side reactions and intermediate production during C–N coupling and improvement in urea electrosynthesis are based on the operational conditions. Our group has been investigating urea synthesis by plasmon-assisted N_2_ and CO_2_ coelectrolysis using silver nanoparticles (Ag NPs) deposited over heterojunctions, Ag@BiVO_4_/BiFeO_3_.^34^ We investigated the electrochemical behavior of N_2_ and CO_2_ coelectrolysis during the excitation of plasmon bands of Ag NPs. We observed through different experiments, like linear sweep voltammetry and controlled potential electrolysis in dark and localized surface plasmon resonance (LSPR) conditions, that N_2_ and CO_2_ are activated to the same extent under LSPR, leading to urea (Figure 4a–d).

**Figure 4:**
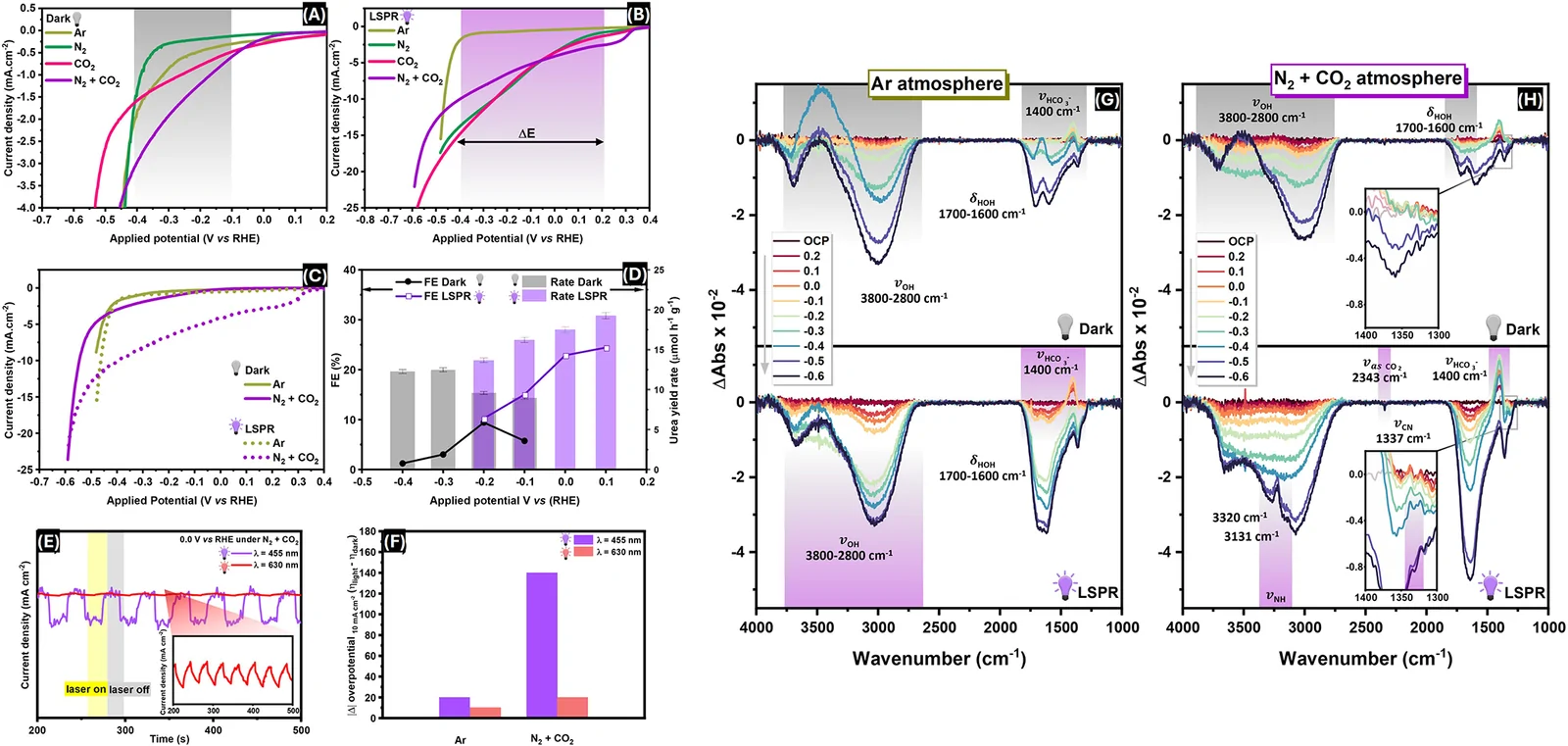
Linear sweep voltammetries in 0.2 mol L^–1^ KHC_3_ at 10 mV s^–1^ (a) in the absence of light (dark) and (b) under LSPR conditions (laser irradiation λ = 455 nm). (c) Comparison between dark and LSPR conditions, (d) Faradaic efficiencies and urea yield rates after controlled potential electrolysis (2 h) under N_2_ + CO_2_, (e) photocurrent analysis under an N_2_ + CO_2_ atmosphere at λ = 455 and λ = 630 nm, (f) bar depicting the wavelength dependent in overpotentials under Ar and N_2_ + CO_2_, (g) *in situ* FTIR-RAS experiments under an Ar atmosphere in the absence of light and LSPR conditions, and (h) under an N_2_ + CO_2_ atmosphere in the absence of light and LSPR conditions, using Ag@BiVO_4_/BiFeO_3_ as a catalyst. Adapted from ref 34, with permission from Elsevier, Copyright 2025.

Interestingly, in a deep analysis of photocurrent experiments comparing Ar and N_2_ + CO_2_ atmospheres, it was observed that only in the last condition, improvements in the current density were observed (Figure 4e). Such behavior suggests that the LSPR increment results in HER suppression, as confirmed by the overpotential necessary to reach 10 mA cm^–2^ (Figure 4f). Experiments of *in situ* FTIR-RAS, Figure 4g,h, also confirmed that the increment of LSPR on the electrochemical process led to a limited amount of H_2_O over the catalyst surface, controlling the HER and consequently enhancing the selectivity for electrochemical C–N coupling.^34^ Such observations at LSPR conditions are a result of local effects, which include the formation of hot carriers, enhancement of the near-field, and local temperature, that need to be explored in detail.^34^

Other operational conditions that have been investigated are the comparison between conventional H-type cells, flow cells, and membrane electrode assembly cells (MEA). Despite the H-cell being the most used configuration with easy access, some drawbacks are associated, which include the effect of operational parameters like temperature, pH, and stirring; the effect of ion membrane exchange; the interface-blocking effect; the gas solubility and diffusion, especially in the case of N_2_; and the limited proton supply.^40,54^ Furthermore, due to such limitations, low current densities and FE and urea yield rates are generally achieved. In particular, the low solubility of gaseous reactants in aqueous electrolytes presents a critical challenge for the C–N coupling reactions. Since CO_2_ solubility remains around 33.8 mM and that of N_2_ around 0.65 mM, and considering that both species must first dissolve into the electrolyte and subsequently diffuse toward the catalyst surface, the substrate availability is one bottleneck.^40,55,56^ Consequently, the reaction rate can be limited by reactant availability rather than by intrinsic catalytic activity, which may lead to underestimation of the real catalytic performance of the materials investigated in H-type cells. Flow cells and MEA cells have been pointed out as promising alternatives to the H-cell in C–N coupling; however, few studies have been reported until now considering such an approach.^40,57–62^ The implementation of flow-cell systems is expected to modify the catalytic performance of materials previously evaluated in H-type cells by improving the reactant transport and increasing the local concentration of gaseous species at the electrode/electrolyte interface. These improvements can potentially enhance C–N coupling pathways, resulting in increased Faradaic efficiency (FE) and urea yield rates. Such improvements in the flow cells and MEA cells are closely related to the circulation of reactants, resulting in a reduction in the mass transfer limitations, like the enhancement in the concentration of N_2_ near the electrode surface.^40,63^

In view of the aspects mentioned, Table 1 summarizes recent achievements in N_2_ and CO_2_ coelectrolysis for C–N coupling.

**Table 1:** Catalysts Reported for Urea Electrosynthesis for N_2_ and CO_2_ Coelectrolysis under Different Conditions

Electrocatalyst^a^	Nitrogen source	Urea yield rate	Potential Applied (V_RHE_)	FE (%)	refs
Fe–N–C	N_2_	0.156 mmol g^–1^ h^–1^	–0.3 V	2.13	^64^
Bi_2_S_3_/N-RGO	N_2_	4.4 mmol g^–1^ h^–1^	–0.5 V	7.5	^65^
Pd_1_Cu_1_/TiO_1_-400^b^	N_2_	3.36 mmol g^–1^ h^–1^	–0.4 V	8.92	^66^
Ag@BiVO_4_/BiFeO_3_	N_2_	8.9 μmol h^–1^ g^–1^	–0.2	9.52	^34^
Sb*_x_* Bi_1–_ *_x_* O_γ_	N_2_	307.97 μg mg_cat_^–1^ h^–1^	–0.3 V	10.9	^67^
Pt_1_Cu_1_–TiO_2_	N_2_	51.71 mol mol_Pt+Cu_^–1^ h^–1^	–0.5 V	11.53	^68^
Bi–BiVO_4_	N_2_	5.91 mmol g^–1^ h^–1^	–0.4 V	12.55	^69^
CuPc NTs	N_2_	143.47 μg mg_cat_^–1^ h^–1^	–0.6 V	12.99	^70^
Cu–W_18_O_49_@ZIF-8	N_2_	1.33 mmol g^–1^ h^–1^	–0.9 V	16.10	^71^
CoPc–MoS_2_	N_2_	175.6 μg mg_cat_^–1^ h^–1^	–0.7 V	15.12	^72^
BiFeO_3_/BiVO_4_	N_2_	4.94 mmol g^–1^ h^–1^	–0.4 V	17.18	^38^
Ni_3_(BO_3_)_2_-150	N_2_	9.70 mmol g^–1^ h^–1^	–0.5 V	20.36	^41^
InOOH-100	N_2_	6.85 mmol g^–1^ h^–1^	–0.4 V	20.97	^42^
Ru–Pd/WO_3_/MXene	N_2_	227 μg mg_cat_^–1^ h^–1^	–0.6 V	23.7	^73^
CoN_3_–Co_AC_/NC	N_2_	20.83 mmol g^–1^ h^–1^	–0.4 V	23.73	^74^
Ag@BiVO_4_/BiFeO_3_ ^c^	N_2_	19.2 μmol h^–1^ g^–1^	+0.1 V	24.26	^34^
V_N_-Cu_3_N-300	N_2_	81 μg cm^–2^ h^–1^	–0.4 V	28.7	^75^
ZnMn–N, Cl	N_2_	4 mmol g^–1^ h^–1^	–0.3 V	28.7	^76^
CeO_2_/Co_3_O_4_	N_2_	5.81 mmol g^–1^ h^–1^	–0.2 V	30.05	^77^
MoP	N_2_	12.4 μg mg_cat_^–1^ h^–1^	–0.35 V	36.5	^78^
Co–PMDA-2-mbIM	N_2_	14.47 mmol g^–1^ h^–1^	–0.5 V	48.97	^79^
MAF-201^d^	N_2_	84 mmol g^–1^ h^–1^	2.0 V^e^	65.5	^80^

a H-cell used in the experiments.

b Flow cell used in the experiments.

c Under plasmonic conditions.

d Membrane electrode assembly cells.

e Cell Voltage.

Considering the data reported in Table 1, it is possible to observe that in most of the studies using different catalysts in the N_2_ + CO_2_ coelectrolysis, the maximum FE achieved ranges below 50% as a result of the challenges reported in this section, and shows that improvements need to be made.

## 3 Electrocatalytic C–N Coupling from NO_3_^–^ and CO_2_

In contrast to N_2_, which is highly abundant in the atmosphere and is not considered a pollutant, the widespread presence of nitrate (NO_3_^–^) largely arises from anthropogenic activities, making it an issue of increasing environmental concern. Some of these anthropogenic activities are related to intensive agriculture and untreated wastewater.^81^ Studies have demonstrated that NO_3_^–^ species contribute to more than 80% of the dissolved inorganic nitrogen in water bodies.^82^ Such presence in water bodies has been raising concerns since NO_3_^–^ is considered a problem related to water quality and also public health problems. In fact, higher levels of NO_3_^–^ in water lead to improvements in the eutrophication processes in water bodies, which is a problem for aquatic ecosystems.^83,84^ Moreover, concerning aspects of public health, excessive amounts in the blood can be converted into other nitrogenous species, including NO_2_^–^, as a result of the activities of some bacteria. As a result of this, the oxygen of N-rich species can bind to red blood cells, causing diseases such as methemoglobinemia and other adverse biological effects, including mutagenesis, carcinogenesis, and teratogenicity.^82,85,86^

Regarding these drawbacks of high amounts of NO_3_^–^ in the environment, it can be considered as an important nitrogen source to produce urea.

During NO_3_^–^ and CO_2_ coelectrolysis, the same premises of N_2_ and CO_2_ coelectrolysis are applied; that is, the NO_3_^–^ and CO_2_ species need to adsorb over the catalyst surface near enough to be further activated and result in C–N coupling. However, in NO_3_^–^ and CO_2_ coelectrolysis, the main challenges are slightly changed. First, NO_3_^–^ presents lower activation barriers and stability within the energy necessary to break the N=O of 204 kJ mol^–1^.^22^ Such values are around three times lower than the energy necessary to break N≡N from N_2_ (941 kJ mol^–1^).^39^ If, on the one side, the thermodynamic barrier is pushed to lower values considering NO_3_^–^ as a nitrogen source, the possibility of different coordination modes via one or two oxygens, associated with its activation process involving multiple proton-coupled electron transfers, drives to a highly complex distribution of intermediates through the following reactions^35,52,87–89^

i $${NO}_{3}^{-}+2H^{+}+2e^{-}⇌{NO}_{2}^{-}+H_{2}O$$

ii $${NO}_{3}^{-}+3H^{+}+2e^{-}⇌{NHO}_{2}+H_{2}O$$

iii $${NO}_{3}^{-}+4H^{+}+3e^{-}⇌NO+2H_{2}O$$

iv $${NO}_{3}^{-}+7H^{+}+6e^{-}⇌{NH}_{2}OH+{2H}_{2}O$$

v $${NO}_{3}^{-}+9H^{+}+8e^{-}⇌{NH}_{3}+3H_{2}O$$

vi $${NO}_{3}^{-}+10H^{+}+8e^{-}⇌N_{2}O+{5H}_{2}O$$

vii $$2{NO}_{3}^{-}+12H^{+}+10e^{-}⇌N_{2}+6H_{2}O$$

The consequence of this complex distribution of possible intermediates and products resulting from NO_3_^–^ activation adds a layer of complexity to the control of different steps during NO_3_^–^ and CO_2_ coelectrolysis, affecting the selectivity for C–N coupling (Figure 5a). Regarding this, it is important to consider that not all of the intermediates formed are supposed to lead to C–N coupling. A representative example in which specific intermediates are required to promote C–N coupling to yield urea is reported in the work of Furuya and colleagues using a Cu-loaded gas diffusion electrode.^25^ In their study, in the presence of NO_3_^–^/NO_2_^–^ and CO_2_, in addition to urea formation, other products/intermediates like NH_3_, HCOOH, and CO were observed. This raised the question of whether it would be possible to produce urea starting from different reactants, like CO/NO_3_^–^/NO_2_^–^ or CO_2_/NH_3_, as in the Bosch–Meiser process. However, considering these intermediate conditions, the only products observed from the catalytic process were NH_3_, CO, and HCOOH, with no urea detected. Despite being related to NO_2_^–^ and CO_2_ coelectrolysis, other reports in the literature observed the same behavior using zinc oxide rich in oxygen vacancies (ZnO-V_o_). The authors also observed that replacing NO_2_^–^ with NH_3_ or CO_2_ with CO during coelectrolysis does not lead to urea formation.^90^ This shows that in both cases, C–N coupling only occurs when both species are simultaneously reduced.^25^

**Figure 5:**
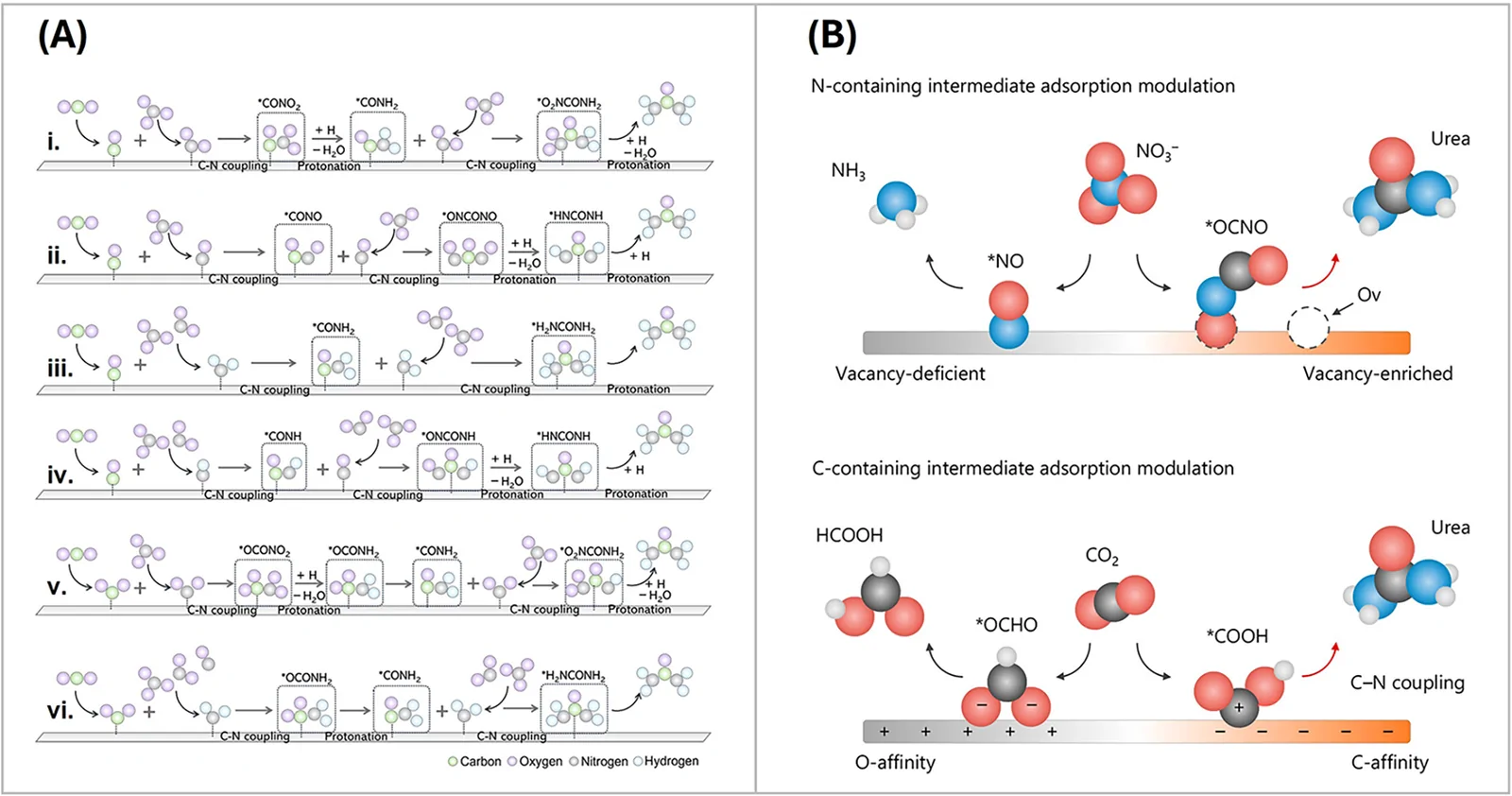
(a) Mechanism diagram of CO_2_ and NO_3_^–^ activation and the C–N coupling process considering the intermediates (i) *CO + *NO_2_, (ii) *CO + *NO, (iii) *CO + *NH_2_, (iv) *CO + *NH, (v) *CO_2_ + *NO_2_, and (vi) *CO + *NH_2_ pathway. Adapted from ref 35. Available under CC BY 4.0. Copyright 2025, Chen, Published by John Wiley-VCH GmbH. (b) Intermediate adsorption modulation for electrocatalytic urea synthesis. (a) (Top) N-containing intermediate adsorption modulation via the construction of a vacant site; (bottom) C-containing intermediate adsorption modulation via surface charge regulation. Reproduced from ref 39. Copyright 2023, American Chemical Society.

Concerning the control between the activation and intermediates formed from NO_3_^–^ and CO_2_ coelectrolysis, studies have focused on the development of catalysts able to activate both species, managing the complex intermediates and species. Based on the previously reported ZnO-V_o_, Wang and colleagues have been putting efforts into the synthesis of different oxygen vacancy CeO_2_ structures and evaluating their contribution toward C–N coupling (Figure 5b­(top)).^91^

Considering the different structures of CeO_2_, pristine and CeO_2_-V_o_, the authors observed that a balance between the amount of oxygen vacancies produced leads to an improvement in urea production through C–N coupling.^91^ A deep series of analyses revealed important behavior across the CO_2_ and NO_3_^–^ species. First, the insertion of oxygen vacancies in CeO_2_-O_v_ led to an improvement in the adsorption of CO_2_, with low activity for the hydrogenation of NO_3_^–^ to NH_3_. The authors pointed out that apparently the oxygen vacancies stabilize the *NO and *CO intermediates to produce *OCNO intermediates, confirmed by *in situ* experiments of sum frequency generation spectroscopy (SFG), instead of proceeding through NO_3_^–^ hydrogenation or formation of carbon monoxide (CO). The opposite behavior is observed for the pristine-deficient oxygen vacancy CeO_2_, in which the catalyst is prone to hydrogenate NO_3_^–^ to NH_3_ with no observation of *OCNO. Furthermore, in the theoretical calculations, the kinetic barrier to produce the *OCNO is almost half lower in CeO_2_-O_v_ (0.27 eV) compared to that in pristine CeO_2_ (0.52 eV) one.^91^ These catalyst modifications allow precise control over reaction intermediates, enabling a concerted activation of NO_3_^–^ and CO_2_, providing key reaction intermediates driving C–N coupling.

The control of intermediates produced in C–N coupling is not only restricted to NO_3_^–^ activation. The control of species derived from CO_2_ also plays an important role in C–N coupling. In another work of Wang and colleagues, the authors investigated how catalyst tuning could interfere with the CO_2_ adsorption and activation during NO_3_^–^ and CO_2_ coelectrolysis (Figure 5b­(bottom)).^92^ The authors produced catalysts based on CuIn alloys supported onto carbon in different ratios of Cu:In, giving Cu_*x*_In_100–x_-C. This approach modulates the surface by regulating the adsorption of CO_2_ in different modes, that is, C-bonded or O-bonded. In this study, it was observed that in catalysts with high Cu-content (*e*.*g*., Cu_97_In_3_–C), the catalyst is negatively charged, in which the sites act as Lewis basic sites and CO_2_ is C-bonded at the surface. Otherwise, at low Cu-contents (*e*.*g*., Cu_30_In_70_–C), the catalyst is positively charged and the sites act as Lewis acid sites with CO_2_ being O-bonded. Such differences in the adsorption of CO_2_ lead to the formation of different intermediates and products. Under conditions in which only CO_2_ is present, when CO_2_ is C-bonded, the intermediate formed is *COOH, and the authors observed the formation of CO as a product; in contrast, when it is O-bonded, *OCHO is formed, and formate is the product. In the presence of NO_3_^–^, the authors demonstrated that C–N coupling is preceded by the formation of intermediate *COOH, reflected in higher values of urea produced, in contrast with the lower ones when *OCHO is involved.^92^ In summary, this reveals that CO_2_ C-bonded intermediates are more prone to C–N coupling than the O-bonded intermediates.

Another approach to the design of catalysts that has been studied to tune the intermediates during the C–N coupling involves spin-polarized catalysts. In the study of Zeng and collaborators, the authors have demonstrated that catalysts based on PdCu lead to the hydrogenation of NO_3_^–^ through NO–H bonds, which is further converted into NH_3_ and is a dead pathway for urea electrosynthesis through NO_3_^–^ + CO_2_ coelectrolysis. However, adding a ferromagnetic species, like Co, gives PdCuCo, and the hydrogenation of NO_3_^–^ proceeds to the ON–H bond, which further leads to C–N coupling. The values for yield rates for urea obtained for the spin-polarized catalyst PdCuCo are higher than the nonspin-polarized PdCu, demonstrating that this pathway is a promising approach to tune the intermediate and, consequently, leads to C–N coupling.^93^

Similar to the example of N_2_ and CO_2_ coelectrolysis, in which the operational conditions can tune C–N coupling, in the NO_3_^–^ and CO_2_ coelectrolysis, efforts have been made to investigate it. In the study of He and collaborators, the authors have investigated NO_3_^–^ and CO_2_ coelectrolysis in a static and conventional coelectrolysis against pulsed coelectrolysis.^94^ Such an approach has as a goal to promote local variations at the interface of the electrode/electrolyte, which includes enhancement of the NO_3_^–^/CO_2_ concentrations, as well as the pH modulation to suppress the local proton concentration and improve C–N coupling. The authors observed that in the static mode, considering the different potentials applied, the maximum FE reached was 19.16% at −0.8 V vs RHE using Fe-TTP/CNT (iron tetraphenyl porphyrin supported over carbon nanotubes). By employing pulsed potential coelectrolysis, with alternating potentials of −0.2 V and −0.8 V vs RHE every 2 s, the authors achieved a FE of 27.70% for urea, proving the beneficial role of dynamic potential modulation.^94^ Such an approach was also applied to commercial catalysts, ZnO and CuPd alloys, in which, under static conditions, the maximum FE achieved for urea, respectively, were 30.72% and 40.29%, in contrast to that achieved for the pulsed ones of 54.83% and 70.36%, respectively. Through different techniques, the authors demonstrated that in pulsed potential electrolysis, the two factors were improved: the enhancement in the concentration of substrates in the interface electrode–electrolyte and the reduction of the local pH.^94^ In fact, as the concentration of substrates increases, a lower amount of water molecules at the interface is expected, as well as the local pH hindering the HER.

In Table 2, it is possible to observe that different catalysts under different conditions present different FEs and urea yield rates when NO_3_^–^ is used as the source during C–N coupling. It is important to point out that, comparing NO_3_^–^ and N_2_ as nitrogen sources, the first one presents higher FE, with values reaching almost 80%, in contrast to those for N_2_, in which the maximum achieved ranges around 50%. However, the potential applied to achieve such an FE when NO_3_^–^ is the nitrogen source is higher compared to that of N_2_.

**Table 2:** Catalysts Reported for Urea Electrosynthesis for NO_3_^–^ CO_2_ Coelectrolysis under Different Conditions

Electrocatalyst^a^	Nitrogen Source	Urea yield rate	Potential Applied (V_RHE_)	FE (%)	refs
Cu@Zn	NO_3_^–^	7.29 μmol cm^–2^ h^–1^	–1.02 V	9.28	^95^
Fe^II^–Fe^III^OOH@BiVO_4_	NO_3_^–^	13.8 mmol h^–1^	–0.8 V	11.5	^76^
XC72R-AuPd	NO_3_^–^	204.2 μg mg^–1^ h^–1^	–0.5 V	15.6	^96^
Fe(a)@C–Fe_3_O_4_/CNTs	NO_3_^–^	1341.3 μg mg_cat_^–1^ h^–1^	–0.65 V	16.5	^97^
FeNi/NC	NO_3_^–^	496.5 μg mg_cat_^–1^ h^–1^	–0.9 V	16.58	^98^
Cu_2_O	NO_3_^–^	62.4 mmol g^–1^ h^–1^	–1.5 V	17.7	^99^
F–CNT-300	NO_3_^–^	6.36 mmol g^–1^ h^–1^	–0.65 V	18.0	^100^
Mo–PdIn BNRs	NO_3_^–^	1016.49 mg g^–1^ h^–1^	–0.4 V	18.42	^101^
Cu–MoSe_2_	NO_3_^–^	1235 μg mg_cat_^–1^ h^–1^	–0.6 V	23.43	^102^
O–Bi_m_/CuO_*x*_	NO_3_^–^	2180.3 μg mg_cat_^–1^ h^–1^	–0.6 V	23.5	^103^
H–PdZn	NO_3_^–^	314.17 μg mg_cat_^–1^ h^–1^	–0.5 V	24.39	^104^
Fe–TPP/CNTs^b^	NO_3_^–^	53 μg mg^–1^ C^1–^	–0.2 V/–0.8 V	27.7	^94^
Cu–GS-800	NO_3_^–^	1800 μg mg_cat_^–1^ h^–1^	–0.9 V	28	^105^
Zn nanosheets	NO_3_^–^	39.3 mmol g^–1^ h^–1^	–0.8 V	31.8	^101^
GB-rich Bi	NO_3_^–^	4.6 mmol mg^–1^ h^–1^	–0.4 V	32.0	^106^
Mo–PCN-222(Co)	NO_3_^–^	844.11 mg g^–1^ h^–1^	–0.4 V	33.9	^107^
SrCo_0.39_Ru_0.61_O_3−δ_	NO_3_^–^	1522 μg mg_cat_^–1^ h^–1^	–0.7 V	34.1	^108^
TiN_0.3_	NO_3_^–^	1488 μg mg_cat_^–1^ h^–1^	–0.72 V	43.1	^109^
Cu_1_Au_8_@CeO_2_	NO_3_^–^	813.6 μg mg_cat_^–1^ h^–1^	–0.94 V	45.2	^110^
Ag–CuNi(OH)_2_	NO_3_^–^	25.6 mmol g^–1^ h^–1^	–0.5 V	46.1	^106^
CoPc–COF@TiO_2_ NTs	NO_3_^–^	1205 μg cm^–2^ h^–1^	–0.6 V	49	^111^
6Å–Cu	NO_3_^–^	7541.9 μg mg_cat_^–1^ h^–1^	–0.41 V	51.97	^112^
In(OH)_3_–S	NO_3_^–^	533.1 μg mg_cat_^–1^ h^–1^	–0.6 V	53.4	^113^
V_O_-S-IO-6	NO_3_^–^	910.4 μg mg_cat_^–1^ h^–1^	–0.6 V	60.6	^114^
Pd_4_Cu_1_–FeNi(OH)_2_	NO_3_^–^	436.9 mmol g^–1^ h^–1^	–0.5 V	66.4	^115^
FeNC–Fe_1_N_4_/C	NO_3_^–^	30.3 mmol g^–1^ h^–1^	–0.6 V	66.5	^116^
PdCu/CBC	NO_3_^–^	763.8 μg mg_cat_^–1^ h^–1^	–0.5 V	69.1	^117^
CuWO_4_	NO_3_^–^	98.5 μg mg_cat_^–1^ h^–1^	–0.2 V	70.1	^118^
CuPd_1_Rh_1_-DAA	NO_3_^–^	53.2 mmol g^–1^ h^–1^	–0.5 V	72.1	^119^
Ru–Cu_9_Bi/CNT	NO_3_^–^	40.0 mmol g^–1^ h^–1^	–0.4 V	75.6	^120^
CuSiO_*x*_ ^b^	NO_3_^–^	1606.1 μg mg_cat_^–1^ h^–1^	–0.6 V	79.01	^121^
CuInS _2_ ^c^	NO_3_^–^	3182.7 μg mg_cat_^–1^ h^–1^	–0.7	24.9	^122^
TiO_2_–C^c^	NO_3_^–^	43.4 mmol g^–1^ h^–1^	–0.9	42.3	^50^

a H-cell used in the experiments.

b Pulsed conditions.

c Flow cell.

## 4 Comparative Analysis: N_2_ vs NO_3_^–^ in Urea Electrosynthesis with CO_2_ Coelectrolysis

Considering the previously mentioned aspects in relation to N_2_ and NO_3_^–^ in the coelectrolysis with CO_2_, it is possible to establish some relationships approaching chemical, practical, and environmental factors, as summarized in Table 3.

**Table 3:** Comparison of Different Aspects Considering N_2_ and NO_3_^–^ as Nitrogen Sources during the Coelectrolysis with CO_2_ for Urea Electrosynthesis

Criteria	N_2_	NO_3_^–^
Chemical reactions	N_2_ + CO_2_ + 6 H^+^ + 6e^–^ → CO(NH_2_)_2_ + H_2_O	2 NO_3_^–^ + CO_2_ + 18 H^+^ + 16e^–^ → CO(NH_2_)_2_ + 7 H_2_O
Dissociation energy^a^	941 kJ mol^–1^ (N≡N)	204 kJ mol^–1^ (N=O)
Solubility in aqueous electrolytes	Low (0.66 mM^40,56^ )	High
Activation	Hard	Easy/Moderate
C–N coupling mechanism	Mostly based on one step	At least two steps regarding NO_3_^–^ activation
Catalysts	Requires a highly specialized approach to overcome activation barriers	Requires a highly specialized approach to overcome selectivity
Side products/selectivity	Moderate, NH_3_, H_2_, CO, HCOOH	Low, NH_3_, NO_2,_^–^ NO, HNO_3_, N_2_O, N_2_, H_2_, CO, HCOOH (possible contamination in the final product)
Faradaic Efficiency (FE) and urea yield rates	Low	Moderate
Availability	High (in atmosphere)	High (in solution)
Sustainability	High, integrated with the circular economy of CO_2_	Moderate (strongly dependent on the source), acts in the removal of contaminants

a CO_2_ energy dissociation = 806 kJ mol^–1^ (C=O).

Considering the chemical aspects of both nitrogen sources in Table 3, N_2_ and NO_3_^–^ in the coelectrolysis with CO_2_, the systems based on NO_3_^–^ benefit from more favorable thermodynamics and reaction kinetics for nitrogen activation. The energy dissociation for the cleavage of the N≡N triple bond in N_2_ requires substantial energy input, leading to sluggish activation and low reaction rates. In contrast, NO_3_^–^, which is already in an oxidized form, requires less energy to dissociate the (N=O) bond, facilitating its reduction. However, this advantage comes at the expense of increased complexity in proton and electron management, as NO_3_^–^ reduction involves multiple proton-coupled electron transfer steps and a wide range of intermediates. Additionally, the mass transport considerations differ significantly between the two systems: while NO_3_^–^ is highly soluble in aqueous electrolytes and can be supplied at molar concentrations, N_2_ has low solubility (∼0.66 mM). Therefore, N_2_ + CO_2_ coelectrolysis is primarily limited by thermodynamic and mass transport constraints, whereas NO_3_^–^ + CO_2_ systems are more challenged by the reaction pathway complexity and selectivity control.

This qualitative trend is corroborated quantitatively by the data sets compiled in Tables 1 and 2. Among the 22 entries of electrocatalysts applied in the N_2_ + CO_2_ coelectrolysis route in Table 1, the reported FE values vary between 2.1 and 65.5%, with only 2 entries exceeding 40% of FE. Both outliers deviate significantly from the experimental framework adopted by the remaining studies and should therefore be interpreted separately. MAF-201 was evaluated in an MEA cell operated at a fixed cell voltage rather than under RHE-referenced H-cell conditions, whereas Co-PMDA-2-mbIM employs a Co-based coordination framework with engineered pore confinement that differs fundamentally from the catalyst architectures represented in the rest of the data set. As a result, neither system is directly comparable. In contrast, the 33 entries for the NO_3_^–^ + CO_2_ coelectrolysis of catalysts compiled in Table 2 vary in a wider and higher range of FE between 9.3 and 79.0%, with approximately one-third of the entries (10) surpassing FE in 50%. Notably, the five highest-performing catalysts for NO_3_^–^ + CO_2_ coelectrolysis are all Cu-containing bimetallic or multimetallic systems. Their consistent performance highlights the importance of multifunctional active sites that can simultaneously promote *NO*_x_* and *CO adsorption and activation, supporting efficient C–N coupling beyond the effects of the overpotential alone. This reinforces, on a quantitative basis, the mechanistic argument above that selectivity control, rather than activation, is the true bottleneck for NO_3_^–^ + CO_2_ coelectrolysis. These statistical differences collectively indicate that despite the remaining challenges in product selectivity, the NO_3_^–^ + CO_2_ route has reached a significantly higher level of catalytic performance than the direct N_2_ + CO_2_ pathway, reflecting the fundamentally distinct reaction landscapes governing the two processes.

The catalyst design requirements also differ between the two systems. Regarding N_2_ + CO_2_ coelectrolysis, catalysts must be highly specialized to enable efficient adsorption and activation of the inert N≡N bond to overcome the activation barriers and stabilize key intermediates along the reaction pathway. Such behavior often demands tailored active sites with precise electronic interactions to facilitate N_2_ activation. Otherwise, for NO_3_^–^ + CO_2_ coelectrolysis, the primary challenge lies not in activation, but in controlling selectivity. Given the high reactivity of NO_3_^–^ and the multiple possible proton-coupled electron transfer pathways, a wide range of intermediate and side reactions can occur, leading to undesired products and intermediates. Therefore, catalysts for NO_3_^–^ activation must be designed to selectively stabilize specific intermediates and suppress competing pathways, ensuring efficient C–N coupling to yield urea. Such aspects in the overall results in a mismatch in the optimal applied potentials as follows: in the N_2_ + CO_2_ system, the requirement for highly negative applied potentials to achieve N≡N bond activation must be balanced against the onset of competitive HER and CO_2_ overreduction. For the NO_3_^–^ + CO_2_ system, the more favorable thermodynamic reduction potential of NO_3_^–^ allows operation at less negative potentials, broadening the window for C–N coupling. This potential mismatch dictates that catalysts for the two systems must be designed with very different active site requirements.

These differences are reflected in the overall performance of the two systems, resulting in lower FE and urea yield rates for N_2_ + CO_2_ coelectrolysis compared to NO_3_^–^ + CO_2_ coelectrolysis. Regarding the overall reduction of the two nitrogen sources, N_2_ and NO_3_^–^, during the electrochemical C–N coupling process, it is important to emphasize that additional experiments using isotopically labeled species (^15^N_2_ and ^15^NO_3_^–^) are still required to unequivocally identify and verify the true nitrogen source. Such isotopic labeling studies would provide reliable and conclusive evidence for the origin of the nitrogen incorporated into the final products. Moreover, in the electrochemical reduction of both nitrogen sources, it is essential to consider the influence of the electrolyte composition on the C–N coupling process. To the best of our knowledge, most studies involving N_2_ + CO_2_ coreduction (Table 1) employ KHCO_3_ as a supporting electrolyte due to its ability to act as a CO_2_ reservoir, maintain the availability of carbonaceous species, and buffer the local pH during the reaction. However, for NO_3_^–^ + CO_2_ coupling (Table 2), different electrolyte compositions have been reported without a standardized approach, mainly due to variations in catalyst stability, nitrate concentration, and the influence of electrolyte properties on the formation and stabilization of reaction intermediates. Previous studies have demonstrated that the physicochemical properties of electrolytes, combined with catalyst surface characteristics, play a crucial role in determining electrocatalytic performance and should also be carefully considered in C–N coupling reactions.^19,123^

Furthermore, considering the complexity of the systems during C–N couplings, such as the partial reduction of substrates like N_2_, NO_3_^–^, and CO_2_, giving side products like NH_3_, NO_2_, CO, HCOOH, and others, as well as the H_2_ from the HER and urea, both systems face challenges regarding the separation and purification of all products produced. Then, to be practically competitive, in addition to the studies related to electrochemical C–N coupling, strategies concerning the separation of the products need to be considered. Different products in different phases, *e*.*g*., gas–liquid and solid–gas phases, are fundamentally easier to separate than products in the same phase, like gas or liquid. Considering C–N coupling, most of the products produced, including urea, face important challenges regarding their separation and purification that need to be addressed. Therefore, studies in this way must be concentrated on technologies that include cryogenic distillation, molecular sieves, salt extraction, and membrane separation to isolate the products produced during C–N coupling.^124,125^ Such approaches are necessary for the future scalability of these electrosynthetic routes.

In terms of sustainability and availability perspective, N_2_ and NO_3_^–^ as nitrogen sources present distinct advantages. N_2_ is highly available in the atmosphere, making it an inherently sustainable nitrogen source, particularly when coupled with CO_2_ valorization, considering the circular-carbon economy. In contrast, NO_3_^–^ is primarily available in aqueous systems, and its sustainability depends strongly on its origin, often linked to anthropogenic activities, like agriculture and industrial activities. Nevertheless, NO_3_^–^ reduction can offer an additional environmental benefit by simultaneously enabling value-added chemical production and control of pollutants, positioning NO_3_^–^ as a moderately sustainable but context-dependent nitrogen source in the urea scenario.

## 5 Conclusions and Perspectives

The coelectrolysis of N_2_ + CO_2_ and NO_3_^–^ + CO_2_ has emerged as a promising alternative approach to the conventional urea synthesis process (Bosch–Meiser process). However, these two nitrogen sources differ significantly in terms of reaction pathways, intermediates, selectivity, and substrate availability. A critical comparison of both, considering their fundamental challenges and catalytic requirements, reveals clear trade-offs. While N_2_ represents an intrinsically sustainable nitrogen source, its practical utilization is hindered by its low solubility and the need for highly specialized catalysts capable of overcoming the strong N≡N bond, stabilizing the key intermediate for C–N coupling to yield urea. In contrast, NO_3_^–^ exhibits more favorable thermodynamics and higher achievable reaction rates accomplished by its high solubility; however, it possesses increased reaction pathway complexity and significant selectivity challenges arising from different intermediates involved in its activation.

Despite the progress reported in recent years, both approaches remain far from practical implementation, as both FE and urea yield rates are low. In the future, the integration of insights from different fields, such as catalyst design, electrolyte engineering, *in situ* and *operando* intermediates/catalyst investigation, and operational optimization, like in the use of flow-cells and MEA cells, seems to be the key to a balance between activity and selectivity. Furthermore, the choice of nitrogen source should not rely only on performance metrics but also consider the sustainability and application context, where N_2_ aligns with long-term circular economy goals, while NO_3_^–^ has additional advantages in environmental remediation.

Based on the comparative analysis presented in Section 4, four key conclusions can be highlighted for the field:

(1) For N_2_ + CO_2_ coelectrolysis, progress is currently constrained less by the applied overpotential than by the lack of catalyst designs capable of activating the N≡N bond while suppressing the competing hydrogen evolution reaction. Although two systems reported FE values above 40% (Table 1), one relied on a tailored Co-based coordination framework and the other on a membrane electrode assembly operated at a fixed cell voltage. Their distinct catalyst architectures and operating conditions make it difficult to extract broadly applicable design principles, highlighting that catalyst development for direct N_2_ activation remains at an early stage.
(2) For NO_3_^–^ + CO_2_ coelectrolysis, the bottleneck is selectivity rather than activation: with up to seven distinct N-containing intermediates competing for the same active sites (eqs 1–7, Section 3), catalysts should prioritize the differential stabilization of *NO_*x*_ and *CO intermediates at spatially adjacent but electronically distinct sites. This strategy is already demonstrated by the bimetallic Cu-based catalysts compiled in Table 2.
(3) The choice between N_2_ and NO_3_^–^ as nitrogen feedstock should not be dictated restrictedly by FE alone but by the intended application context: N_2_ + CO_2_ aligns with atmosphere-derived, circular-carbon strategies decoupled from any waste stream, whereas NO_3_^–^ + CO_2_ is best positioned as a value-added remediation route for nitrate-contaminated agricultural and industrial effluents, where feedstock availability, rather than sustainability, is the decisive factor.
(4) Meaningful across-study comparisons will remain compromised until the field adopts standard practices: (i) isotopically labeled experiments (^15^N_2_ and ^15^NO_3_^–^ control experiments) and (ii) consistent cell configuration (H-cell versus flow cell/MEA) when reporting FE.
